# Forecasting Demand for the Typhoid Conjugate Vaccine in Low- and Middle-income Countries

**DOI:** 10.1093/cid/ciy1076

**Published:** 2019-03-07

**Authors:** Frédéric Debellut, Nathaniel Hendrix, Virginia E Pitzer, Kathleen M Neuzil, Dagna Constenla, Naor Bar-Zeev, Anthony Marfin, Clint Pecenka

**Affiliations:** 1 Center for Vaccine Innovation and Access, PATH, Geneva, Switzerland; 2 The Comparative Health Outcomes, Policy, and Economics (CHOICE) Institute, University of Washington, Seattle; 3 Department of Epidemiology of Microbial Diseases, Yale School of Public Health, New Haven, Connecticut; 4 Center for Vaccine Development and Global Health at the University of Maryland School of Medicine, Baltimore, MD; 5 Department of International Health, Johns Hopkins Bloomberg School of Public Health, Baltimore, Maryland; 6 Center for Vaccine Innovation and Access, PATH, Seattle, Washington

**Keywords:** typhoid fever, vaccination, typhoid conjugate vaccine, demand forecasting

## Abstract

**Background:**

The World Health Organization (WHO) released a position paper in March 2018 calling for integration of a novel typhoid conjugate vaccine (TCV) into routine immunization along with catch-up campaigns for children up to age 15. Gavi, the Vaccine Alliance, has committed funding to help resource-constrained countries introduce this vaccine. In this article, the Typhoid Vaccine Acceleration Consortium forecasts demand if WHO recommendations are followed.

**Methods:**

We built a model of global TCV introductions between 2020 and 2040 to estimate the demand of the vaccine for 133 countries. We estimated each country’s year of introduction by examining its estimated incidence of typhoid fever, its history of introducing new vaccines, and any knowledge we have of its engagement with typhoid prevention, including intention to apply for Gavi funding. Our model predicted use in routine infant vaccination as well as campaigns targeting varying proportions of the unvaccinated population up to 15 years of age.

**Results:**

Between 2020 and 2025, demand will predominantly come from African countries, many receiving Gavi support. After that, Asian countries generate most demand until 2030, when campaigns are estimated to end. Demand will then track the birth cohort of participating countries, suggesting an annual routine demand between 90 and 100 million doses. Peak demand is likely to occur between 2023 and 2026, approaching 300 million annual doses if campaign implementation is high.

**Conclusions:**

In our analysis, target population for catch-up campaigns is the main driver of uncertainty. At peak demand, there is some risk of exceeding presently estimated peak production capacity. Therefore, it will be important to carefully coordinate introductions, especially when accompanied by campaigns targeting large proportions of the eligible population.

Global typhoid burden has fallen since the 1990s, incidence appears stable over the past few years [1], and antimicrobials have lowered mortality associated with typhoid fever. However, antimicrobial resistance has become increasingly common, and multidrug-resistant bacteria are increasingly driving epidemics [2–5]. Improved sanitation and water treatment have reduced transmission, but widespread vaccination will be critical to further prevention efforts, especially against antimicrobial resistant typhoid and to prevent outbreaks.

Because neither the live attenuated nor the subunit vaccine can be given to children under 2 years of age [6], for whom typhoid fever causes substantial morbidity and mortality [7], researchers have developed typhoid conjugate vaccines (TCVs) that are safe in children 6 months of age and older and produce an improved immune system response [8]. Based on the recommendation of the Strategic Advisory Group of Experts (SAGE), in 2018 the World Health Organization (WHO) released a position paper calling for greater use of typhoid vaccines with preference given to TCV [1]. Following the WHO position paper, Gavi, the Vaccine Alliance committed funding to support countries in their efforts to introduce TCV [9].

In this analysis, we project demand for TCV in low- and middle-income countries based on the latest WHO recommendations. Demand forecasting will aid in the overall strategic planning for TCV introduction by allowing stakeholders to assess a range of introduction scenarios and related volumes [10]. It can also identify the different factors that influence demand for vaccine. By doing so, demand forecasts can assist in aligning the interests of all the stakeholders—industry, countries, and donors—whose participation is essential to successful vaccination programming.

## METHODS

### Model Overview

We developed a model to estimate demand for TCV in 133 low-, lower-middle-, and upper-middle-income countries between the years 2020 and 2040, assuming WHO recommendations are followed [11]. Because TCV is the first typhoid vaccine eligible to be added to the Expanded Program on Immunization (EPI) schedule, we anticipate that vaccination of 9-month-old infants will form the basis of most countries’ introduction strategies.

WHO also recommends that introduction in EPI be accompanied by a catch-up campaign to vaccinate all children 15 years of age and younger who did not receive TCV in infancy. As this campaign may target all members of this age cohort or only those considered high risk, we examine 6 scenarios with target cohorts for the catch-up campaigns ranging from 0% to 100% of the eligible population.

The model calculates the number of TCV doses distributed in both routine and campaign administration settings while adjusting for 2 additional sources of demand: wastage due to expiration or spoilage; and buffer stock, which is a 1-time purchase of extra vaccine meant to support continued use in case of supply disruptions.

### Model Inputs and Assumptions

Each country’s estimated birth cohort was derived from the medium projection of the 2017 revision of the United Nations (UN) World Population Prospects [12]. We excluded from our analysis countries with a total population less than 100 000 as well as those who are not members of the UN or WHO. As the UN projects populations in 5-year age bands, we interpolated single-year age cohorts from their figures. We did this by dividing the population in a given 5-year age band by 5, then placing that quotient at the middle year of the age band (eg, age 2 for the age band 0–4). We then used a linear slope ([population_age k+5_ – population_age k_] / 5) to estimate the difference in the single year age-based cohorts at a given point in time. Populations less than 2 years of age were assumed to follow the same slope as the change in population from age 2 to age 7.

We used a similar method to interpolate between the UN’s 5-year cross-sectional projections. This method has been fully detailed in another publication [13].

An important uncertainty in the model is the projected year of introduction. In order to estimate when each country would introduce TCV, we were guided by 2 principles: first, that the countries with the highest typhoid incidence would introduce before countries with lower incidence; second, that countries’ recent history of new vaccine introduction is predictive of the year in which they would introduce TCV. Paradoxically, countries with the highest typhoid incidence may be those with the fewest resources to invest in new vaccine introduction, though Gavi support to many countries helps alleviate this challenge. We account for this by utilizing information on the timing of past introductions to guide our estimates. Finally, we adjusted some countries’ final introduction dates from the default to account for current knowledge about their level of interest.

Although researchers have recently developed improved methods for estimating the burden of typhoid fever, 3 sources of incidence data [14–17] were highly variable in many instances (see Supplementary Table 1 for a comparison). Therefore, for each country, we used the median value and classified the 133 countries incidence into 4 tiers of all-age incidence based on standard incidence categories for typhoid fever (Table 1) [18].

**Table 1.** Incidence Categories

Incidence Category	Estimated Median Incidence per 100 000 pyo	Number of Countries
Low	<10	11
Medium	10–99	58
High	100–499	58
Very high	≥500	6

Abbreviation: pyo, person years observed.

We gathered the years in which each country introduced 3 recently available vaccines—pneumococcal conjugate vaccine, rotavirus vaccine, and human papillomavirus vaccine— to estimate each country’s current approach to vaccine-related decision-making [19]. We defined 4 groups according to the number of vaccines introduced by 2018. Countries that had introduced all 3 vaccines were considered early adopters; countries who had introduced 2 vaccines were moderate adopters, whereas late adopters had introduced 1 vaccine, and nonadopters had introduced no vaccines.

We applied an adjustment to this classification based on the average number of years lapsing between Gavi prequalification of the 3 vaccines above and their introduction in each country. If a given country introduced these vaccines within an average of 0 to 3 years after Gavi prequalification, its classification was adjusted 1 category toward early adoption (eg, from late to moderate adoption). On the other hand, if 7 or more years lapsed between Gavi prequalification and the country’s introduction(s), the country’s classification was adjusted toward late adoption (eg, from moderate to late adoption). We then used the matrix in Table 2 to derive the default year of vaccine introduction.

**Table 2.** Default Year of Vaccine Introduction by Burden and Historical Speed of Vaccine Introduction

	Incidence			
	Very High	High	Medium	Low
Early adopter	2021	2022	2027	Not before 2040
Medium adopter	2023	2024	2028	Not before 2040
Late adopter	2025	2026	2029	Not before 2040
Nonadopter	Not before 2040	Not before 2040	Not before 2040	Not before 2040

**Figure 1.**
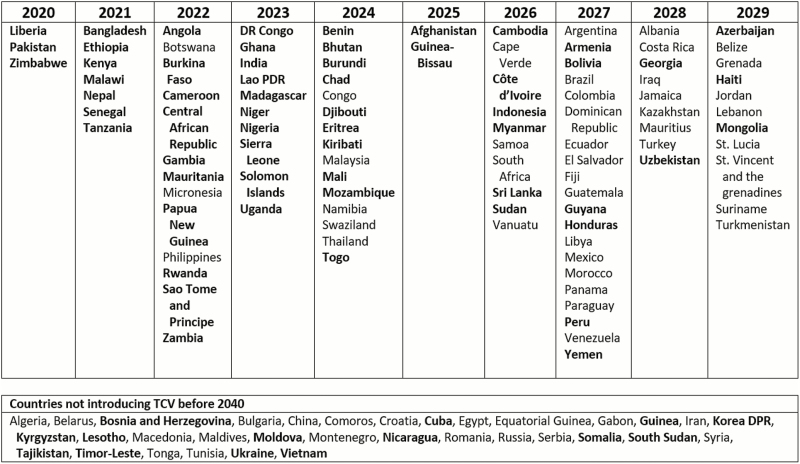
Base case year of introduction. Bold font indicates Gavi eligibility as of 2018. Abbreviation: TCV, typhoid conjugate vaccine.

We applied further adjustments to the default year of TCV introduction based on what is known about certain countries. Some eligible countries have expressed interest in applying for Gavi funding to support early TCV introduction, driven by factors such as the occurrence of recent outbreaks of typhoid fever. We considered these countries early adopters. Next, we advanced the introduction date by 1 year for countries who have participated in typhoid disease surveillance, TCV development, or other typhoid-related activities. Countries that are projected to graduate from Gavi support within the model’s time horizon were assumed to apply for support in their last year of accelerated transition, indicating that they would begin using TCV within the following 18 months. Finally, we considered that countries with low incidence would be unlikely to introduce even with Gavi support due to the low priority presumably assigned to typhoid fever. The final introduction base case is presented in Figure 1, whereas unadjusted default values can be viewed in Supplementary Table 2.

As is common in demand forecasting [13, 20], and considering TCV is likely to be administered between 9 and 12 months of age, we based our estimate of TCV coverage in both routine and campaign settings on each country’s rate of coverage for the first dose of measles-containing vaccine (MCV1) [21]. The most recent observed rate of MCV1 coverage as of June 2018 was carried forward unchanged to the end of the model’s time horizon for both EPI and catch-up campaigns. We assumed that TCV’s availability in EPI slowly increases in a linear fashion rather than achieving full coverage immediately upon introduction. Although data on time to reach maximum vaccine coverage (ie, equal to MCV1) in EPI is limited, it is generally believed that it is proportional to population size. Accordingly, we estimated the number of years needed to reach maximum coverage based on birth cohort size in the year of introduction, as follows:

- ≤15 000 = 1 year
- 15 001 to 500 000 = 2 years
- 500 001 to 1 000 000 = 3 years
- >1 000 000 = 4 years

The population eligible to receive catch-up vaccination is defined as the population aged 9 months to 15 years at the time of TCV introduction. As subnational campaigns may be common, we tested 6 scenarios in which 0%, 10%, 25%, 50%, 75%, or 100% of the age-defined population is targeted in the catch-up campaign. These percentages are then multiplied by each country’s MCV1 rate to define the population actually covered in the campaign.

We assumed that catch-up campaigns would last 1 year for most countries, with those having a birth cohort greater than 2.5 million at time of introduction requiring an additional year. In these larger countries, we assumed that the target population was divided equally between the 2 years of the campaign. Because there is some uncertainty around who will participate in these campaigns, we have also run a scenario in which countries that are not Gavi-eligible in 2020 will not run catch-up campaigns.

For catch-up campaigns, we assumed a wastage rate of 10%, as compared to 15% in routine vaccination. We also assumed that countries would purchase an additional 25% of their estimated annual routine vaccine demand in their year of introduction to account for buffer stock, as suggested by WHO guidance. All model parameters are detailed in Supplementary Table 3.

### Probabilistic Sensitivity Analysis

In addition to the base case scenarios described above, we also ran a probabilistic sensitivity analysis in order to estimate uncertainty in each year’s projected demand. We varied three parameters of the model in 5000 runs of a Monte Carlo simulation: introduction year, percent of eligible population targeted in catch-up campaigns, and coverage. For the year of introduction, we used a uniform distribution plus or minus 2 years from the base case (bounded on the model’s time horizon of 2020 to 2040). We assumed an equal probability of selecting each of the 6 levels of population to be targeted in catch-up campaigns and allowed this percentage to vary by country. Finally, to estimate uncertainty around coverage, we used a normal distribution with mean set at the base case value (ie, MCV1) and a standard deviation of 5 percentage points, bounded between 0 and 1.

Participation in catch-up campaigns is likely to explain a significant amount of the variation seen in the probabilistic sensitivity analysis, because the population from age 1 to 15 is much larger than the infant population that is eligible for EPI. Therefore, we also ran a probabilistic sensitivity analysis while assuming no catch-up campaigns in order to isolate the effect of other variables.

## RESULTS

**Figure 2.**
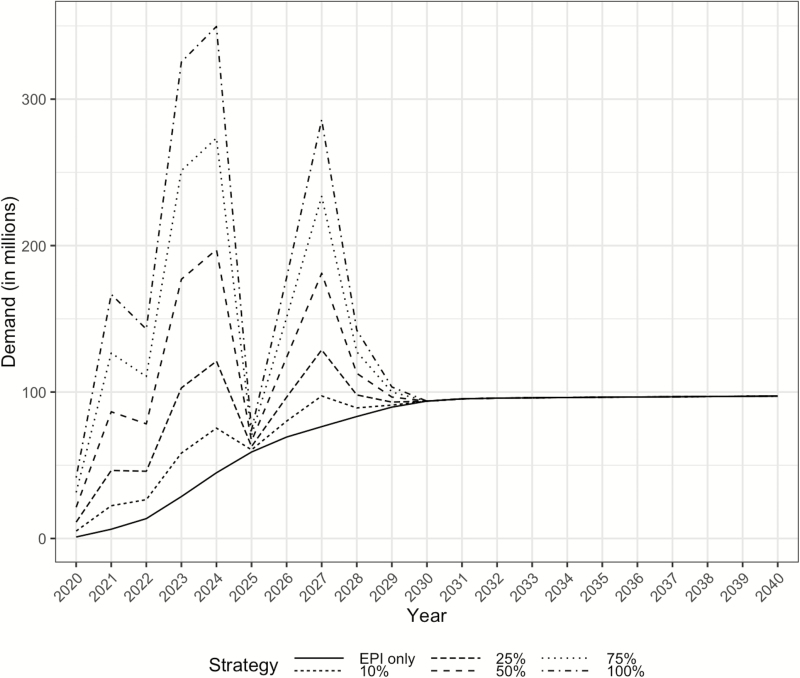
Demand for typhoid conjugate vaccine by year, according to six scenarios in which different percentages of the 9-months to 15-year-old population are targeted in catch-up campaigns. Abbreviation: EPI, Expanded Program on Immunization.

Assuming that 25% or more of eligible children are vaccinated in catch-up campaigns, demand for TCV accelerates quickly between 2020 and 2023 and remains high through 2024 (Figure 2). If, for example, 25% of eligible children are targeted for catch-up campaigns alongside routine administration, peak demand is approximately 100 million doses. Peak demand approximately doubles if 50% of eligible children are targeted for catch-up campaigns. The first 4 years of introduction are heavily dominated by catch-up campaigns in African countries, many of whom receive Gavi support. Starting in 2026, nations in the South-East Asia Region account for most of the projected demand. The same year, non-Gavi and Gavi-graduate countries are projected to begin generating the most demand. The scenarios converge near 94 million annual vaccines per year in 2030 after all catch-up campaigns end. Aggregated results are in Table 3, whereas country-level results are available in Supplementary Table 4. The scenario in which non-Gavi countries do not run campaigns found that campaign-based demand was reduced by an average of 55% per year, regardless of the size of the target population.

**Table 3.** Annual Demand (in Thousands) by Usage Scenario

	2020	2023	2025	2027	2030
EPI + EPI wastage	835	26 926	58 923	75 857	93 784
Buffer stock	181	1696	111	509	0
Campaign + campaign wastage (based on relative coverage)					
10%	4060	29 677	1507	20 959	0
25%	10 150	74 192	3766	52 396	0
50%	20 300	148 383	7533	104 793	0
75%	30 451	222 575	11 299	157 189	0
100%	40 601	296 766	15 065	209 585	0

Abbreviation: EPI, Expanded Program on Immunization.

**Figure 3.**
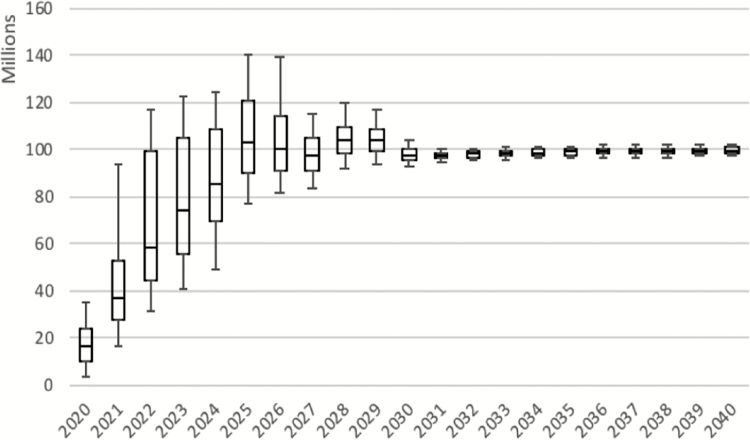
Results of the probabilistic sensitivity analysis. The central box for each year represents the median as well as 25th and 75th percentiles; the “whiskers” represent the 5th and 95th percentile.

The probabilistic sensitivity analysis suggests extremely wide uncertainty intervals in the early years of TCV introduction (see Figure 3). It also suggests that peak demand may occur as late as 2026. Uncertainty in demand is predicted to be significantly reduced starting in 2028 due to fewer introductions (and thus fewer campaigns) among countries with large populations. Of note, the sensitivity analysis was built to express uncertainty on an annual basis rather than as a set of scenarios expressing the trajectory of overall demand. Thus, for example, extremely high demand 1 year may be followed by lower than average demand the following year. It also does not include countries not projected to introduce before 2040. In our scenario analysis examining only factors other than campaign target population, we found that the interquartile range was 69% smaller through 2030, suggesting that other factors explain 31% of the variation seen in the probabilistic sensitivity analysis.

## DISCUSSION

In this article, we used a demand forecasting model to predict 20 years of TCV usage based on WHO recommendations, estimated TCV introduction dates, and recent new vaccine introductions. Our modeling approach, input variables, and assumptions are transparent and reflect the best global data. However, specific country or time-based inferences should be made with caution; therefore, we have also made efforts to quantify the forecast’s uncertainty and some of the major drivers by including a probabilistic sensitivity analysis.

Our model suggests that 3 phases in TCV demand will occur. The first, lasting from 2020 until 2025, shows increasing introduction among Gavi-supported countries, mostly in Africa. The second phase, which will likely last from 2026 until 2029, is characterized by increasing demand from South and Southeast Asian countries. Peak demand may be reached in either the first or second phase, depending on the percent of campaign-eligible individuals targeted in each country. Finally, vaccine demand generated by catch-up campaigns tapers off around 2030. Increases in demand in this phase are projected to be due to increases in the size of birth cohorts rather than new TCV introductions, assuming countries maintain their TCV immunization program as typhoid is controlled. Maintaining immunization programs will be important to prevent future outbreaks and limit antimicrobial resistance.

Our probabilistic sensitivity analysis suggests that there is wide variability in these demand forecasts. Nevertheless, both the base case analysis and the sensitivity analysis predict that demand may increase rapidly within the first 3 to 5 years after initial TCV introductions in 2020. This means that there is likely to be significant value in gaining greater in-depth knowledge about countries’ introduction plans. The 6 scenarios that we used in the base case analysis indicate that the target population for catch-up campaigns is the main driver of uncertainty, although year of introduction would also be valuable to ascertain, especially for large countries.

There are also other sources of variability that we have not been able to include in the model. For example, updated national census information may not accord with current UN population estimates. This is the case with Pakistan, for example, whose application for Gavi funding projected higher routine demand due to a higher projected birth rate than the UN had modeled in their medium variant.

There is currently 1 supplier of TCV, Bharat Biotech International, who can produce 50 million vaccines per year and is working to expand production capacity to 200 million a year after first national introduction [22]. Apart from the potential risks associated with a single supplier, our probabilistic sensitivity analysis suggests that supply constraints may be a concern during the roll-out of TCV if catch-up campaigns are widely used (ie, target more than 50% of the age-defined cohort).

There is only 1 other published article projecting demand for TCVs in low- and middle-income countries [23]. Mogasale et al also project an eventual leveling off of demand near 100 million annual doses; however, unlike our model, theirs did not suggest that introduction was likely to occur in geographically defined phases. In their scenario, modeling EPI and catch-up of high-risk individuals, they found that demand is likely to peak 8 years after the first introductions at just over 200 million doses. They define high-risk individuals as those who live in urban slums or in rural areas without access to safe water [24]. However, due to existing uncertainty around risk factors [25, 26] and the possibility of outbreaks, we have chosen to model the target population of catch-up campaigns as a global percentage rather than a country-specific percentage; this parameter, though, was the greatest contributor to uncertainty in the results of the probabilistic sensitivity analysis.

The model we present is limited in several ways, mostly by our assumptions around introduction dates and planning for campaigns. Introduction decisions, dates, and campaign target populations are both critical and uncertain. Although data on time to achieve maximum coverage are scarce, such data would have helped us to make more accurate projections of the speed with which countries go from initial introduction to maximum coverage. This parameter is likely to be an important determinant of when demand for TCV will peak and should therefore be watched closely. It also remains to be seen whether countries will match the coverage that they have achieved with MCV1. Finally, we did not model epidemics. The recent WHO recommendations encourage deployment of TCV during outbreaks, which may mean that some countries accelerate their introduction.

Future work remains to be done on the predictors of vaccine introduction and coverage, which would facilitate more accurate demand forecasting. Also, further refinements in the measurement of typhoid fever’s burden may be worthwhile, as inaccurate perceptions of disease incidence may have discouraged adoption after previous WHO recommendations [7]. Current estimates, although more robust in methodology, still diverge considerably. This has important implications for when we forecast vaccine introduction to occur in each country. The Typhoid Vaccine Acceleration Consortium anticipates regularly updating the demand forecast, which will provide an opportunity to validate ex post the estimates provided in this article.

## CONCLUSIONS

Our estimate of the annual demand for TCV suggests that it is likely to peak between 2023 and 2026, although there is very high uncertainty around how many doses will be needed at this peak. We find that the period between 2030 and 2040 is likely to be characterized by a “steady state” in which demand remains relatively flat between 90 and 100 million doses per year. Our model suggests that there is some risk of demand exceeding supply in 2023 and 2024, indicating that introductions may need to be carefully coordinated and that advance knowledge of large countries’ introduction plans will be valuable. That said, long-term demand is unlikely to exceed current estimates of production capacity.

Our estimates of the TCV demand that would result from following recent WHO recommendations for typhoid vaccination may be used by public health planners to assess the value of gaining more knowledge around production capacity and of obtaining national commitments to introduction. Policymakers in countries should consider appropriate TCV implementation strategies in advance of licensure.

## Supplementary Data

Supplementary materials are available at *Clinical Infectious Diseases* online. Consisting of data provided by the authors to benefit the reader, the posted materials are not copyedited and are the sole responsibility of the authors, so questions or comments should be addressed to the corresponding author.

Supplementary Table 1

Supplementary Table 2

Supplementary Table 3

Supplementary Table 4

## Notes

***Acknowledgments.*** The authors thank Matthew Blakley, Zoey Diaz, Melissa Ko, Samya Mandal, Vittal Mogasale, and Vinita Vishwanarayan for their review and comments on earlier versions of the model and analysis.

***Financial support.*** This publication is based on research funded in part by grants from the Bill & Melinda Gates Foundation (OPP1151153 and OPP1116967]). N. B.-Z. received grants from Path, Seattle, related to the present work. D. C. received funding from Bill & Melinda Gates Foundation via an Oxford University (Typhoid Vaccine Acceleration Consortium) grant, for the present work.

***Supplement sponsorship.*** This supplement is sponsored by the Center for Vaccine Development and Global Health (CVD) at the University of Maryland School of Medicine.

***Potential conflicts of interest.*** D. C. reports grants from the Bill & Melinda Gates Foundation, outside the submitted work. C. P. received travel support from the Bill & Melinda Gates Foundation to attend a meeting on respiratory syncytial virus maternal immunization. K. M. N. is a member of the World Health Organization Strategic Advisory Group of Experts on Immunization. All other authors report no potential conflicts. All authors have submitted the ICMJE Form for Disclosure of Potential Conflicts of Interest. Conflicts that the editors consider relevant to the content of the manuscript have been disclosed.
