# Supplementary material for: Forecasting Demand for the Typhoid Conjugate Vaccine in Low- and Middle-income Countries
Source: Clin Infect Dis. 2019 Mar 7;68(Suppl 2):S154–60. doi: 10.1093/cid/ciy1076 (PMC6405267; doi:10.1093/cid/ciy1076)
Supplement: Supplementary Table 1 [file ciy1076_suppl_supplement_table_1.docx]

*Supplementary table 1: Median incidence and related burden group*

| Country | Typhoid fever incidence rate per 100,000, all age IHME (GBD 2016) | Typhoid fever incidence rate per 100,000, all age, Antillon (2017) | Typhoid fever incidence rate per 100,000, all age, Mogasale (risk factor adjusted) 2017 | Median incidence rate per 100,000 | Burden group |
| --- | --- | --- | --- | --- | --- |
| Afghanistan | 73.96 | 1,233.49 | 744.26 | 744.26 | Very high |
| Albania | 0.2 | 69.37 |  | 34.78 | Medium |
| Algeria | 35.65 | 27.83 | 13.4 | 27.83 | Medium |
| Angola | 98.95 | 287.06 | 586.17 | 287.06 | High |
| Argentina | 0.29 | 52.23 | 27.43 | 27.43 | Medium |
| Armenia | 0.3 | 14.32 | 10.45 | 10.45 | Medium |
| Azerbaijan | 0.4 | 35.81 | 14.3 | 14.3 | Medium |
| Bangladesh | 545.09 | 1,100.71 | 315.51 | 545.09 | Very high |
| Belarus | 0.11 | 8.65 |  | 4.38 | Low |
| Belize | 1.81 | 132.71 | 33.55 | 33.55 | Medium |
| Benin | 172.21 | 1,925.84 | 410.89 | 410.89 | High |
| Bhutan | 401.95 | 101.27 | 346.49 | 346.49 | High |
| Bolivia | 1.95 | 337.64 | 40.74 | 40.74 | Medium |
| Bosnia and Herzegovina | 0.14 | 6.12 |  | 3.13 | Low |
| Botswana | 2.2 | 128.94 | 175.58 | 128.94 | High |
| Brazil | 1.75 | 98.19 | 30.4 | 30.4 | Medium |
| Bulgaria | 0.29 | 6.9 |  | 3.59 | Low |
| Burkina Faso | 405.49 | 570.78 | 518.22 | 518.22 | Very high |
| Burundi | 105.3 | 1,980.64 | 273.58 | 273.58 | High |
| Cambodia | 334.42 | 646.03 | 352.87 | 352.87 | High |
| Cameroon | 181.75 | 264.51 | 462.83 | 264.51 | High |
| Cape Verde | 107.5 | 30.02 | 306.43 | 107.5 | High |
| Central African Republic | 99.04 | 843.37 | 529.52 | 529.52 | Very high |
| Chad | 225.49 | 904.55 | 560.83 | 560.83 | Very high |
| China | 22.26 | 24.72 | 16.05 | 22.26 | Medium |
| Colombia | 2.1 | 104.28 | 27.87 | 27.87 | Medium |
| Comoros | 76.7 | 56.31 | 272.86 | 76.7 | Medium |
| Congo | 57.79 | 365.1 | 493.37 | 365.1 | High |
| Congo, DR | 104.06 | 1,622.93 | 584.01 | 584.01 | Very high |
| Costa Rica | 0.16 | 63.22 | 23.43 | 23.43 | Medium |
| Côte d'Ivoire | 133.6 | 607.09 | 366.3 | 366.3 | High |
| Croatia | 0.13 |  |  | 0.13 | Low |
| Cuba | 0.27 | 29.44 | 19.28 | 19.28 | Medium |
| Djibouti | 70.72 | 104.46 | 232.75 | 104.46 | High |
| Dominican Republic | 7.03 | 21.65 | 27.73 | 21.65 | Medium |
| Ecuador | 2.57 | 558.87 | 28.91 | 28.91 | Medium |
| Egypt | 33.19 | 22.94 | 13.26 | 22.94 | Medium |
| El Salvador | 3.15 | 30.5 | 34.27 | 30.5 | Medium |
| Equatorial Guinea | 54.23 | 12.36 |  | 33.3 | Medium |
| Eritrea | 124.82 | 516.49 | 326.61 | 326.61 | High |
| Ethiopia | 116.75 | 613.71 | 392.41 | 392.41 | High |
| Fiji | 100.01 | 38.66 | 20.91 | 38.66 | Medium |
| Gabon | 44.44 | 221.34 | 407.63 | 221.34 | High |
| Gambia | 115.71 | 198.98 | 336.24 | 198.98 | High |
| Georgia | 0.3 | 11.15 | 10 | 10 | Medium |
| Ghana | 187.41 | 205.78 | 250.25 | 205.78 | High |
| Grenada | 1.05 | 22.64 | 44.85 | 22.64 | Medium |
| Guatemala | 24.72 | 192.57 | 36.18 | 36.18 | Medium |
| Guinea | 149.93 | 649.11 | 2.72 | 149.93 | High |
| Guinea-Bissau | 28.01 | 268.97 | 436.98 | 268.97 | High |
| Guyana | 3.35 | 144.46 | 28.37 | 28.37 | Medium |
| Haiti | 5.16 | 190.96 | 46.9 | 46.9 | Medium |
| Honduras | 6.63 | 119.65 | 36.74 | 36.74 | Medium |
| India | 499.01 | 81.27 | 438.94 | 438.94 | High |
| Indonesia | 327.97 | 74.1 | 140.15 | 140.15 | High |
| Iran | 30.33 | 14.78 | 338.46 | 30.33 | Medium |
| Iraq | 44.15 | 17.93 | 22.95 | 22.95 | Medium |
| Jamaica | 1.29 | 10.14 | 35.01 | 10.14 | Medium |
| Jordan | 46.88 | 9.03 | 14.66 | 14.66 | Medium |
| Kazakhstan | 0.27 | 27.67 | 248.65 | 27.67 | Medium |
| Kenya | 209.9 | 193.59 | 809.35 | 209.9 | High |
| Kiribati | 129.29 | 596.08 | 21.26 | 129.29 | High |
| Korea, DPR | 9.73 | 496.97 | 14.25 | 14.25 | Medium |
| Kyrgyzstan | 0.3 | 19.46 | 304.43 | 19.46 | Medium |
| Lao, PDR | 320.24 | 838.34 | 406.24 | 406.24 | High |
| Lebanon | 55.84 | 17.3 | 18.45 | 18.45 | Medium |
| Lesotho | 2.94 | 47.67 | 204.71 | 47.67 | Medium |
| Liberia | 160.29 | 1,337.74 | 375.33 | 375.33 | High |
| Libya | 34.81 | 50.35 | 15.4 | 34.81 | Medium |
| Madagascar | 106.35 | 1,252.88 | 382.2 | 382.2 | High |
| Malawi | 89.96 | 174.33 | 298.21 | 174.33 | High |
| Malaysia | 128.59 | 15.54 | 209.02 | 128.59 | High |
| Maldives | 35.36 | 11.39 | 297.42 | 35.36 | Medium |
| Mali | 174.41 | 408.25 | 424.08 | 408.25 | High |
| Mauritania | 203.02 | 175.75 | 379.06 | 203.02 | High |
| Mauritius | 14.93 | 8.32 | 160.48 | 14.93 | Medium |
| Mexico | 10.57 | 53.88 | 26.58 | 26.58 | Medium |
| Micronesia | 116.39 | 228.59 | 15.26 | 116.39 | High |
| Mongolia | 0.68 | 327.81 | 23.23 | 23.23 | Medium |
| Montenegro | 0.13 | 14.74 |  | 7.43 | Low |
| Morocco | 39.59 | 37.43 | 15.55 | 37.43 | Medium |
| Mozambique | 167.96 | 293.55 | 410.47 | 293.55 | High |
| Myanmar | 193.01 | 310.01 | 267.15 | 267.15 | High |
| Namibia | 2.63 | 158.74 | 179.27 | 158.74 | High |
| Nepal | 435.58 | 113.25 | 375.35 | 375.35 | High |
| Nicaragua | 3.16 | 139.47 | 39.72 | 39.72 | Medium |
| Niger | 162.58 | 453.87 | 510.33 | 453.87 | High |
| Nigeria | 197.46 | 605.42 | 451.35 | 451.35 | High |
| Pakistan | 300.81 | 123.24 | 292.56 | 292.56 | High |
| Panama | 2.82 | 41.34 | 29.34 | 29.34 | Medium |
| Papua New Guinea | 256.13 | 7,892.22 | 24.94 | 256.13 | High |
| Paraguay | 1.89 | 94.39 | 34.63 | 34.63 | Medium |
| Peru | 2.5 | 224.07 | 34.79 | 34.79 | Medium |
| Philippines | 216.48 | 878.7 | 310.65 | 310.65 | High |
| Republic of Moldova | 0.01 | 5.86 |  | 2.93 | Low |
| Romania | 0.41 | 18.35 |  | 9.38 | Low |
| Russian Federation | 0.5 | 17.26 |  | 8.88 | Low |
| Rwanda | 100.03 | 344.85 | 316.41 | 316.41 | High |
| Saint Lucia | 3.22 | 46.83 |  | 25.02 | Medium |
| Samoa | 112.67 | 147.82 | 15.55 | 112.67 | High |
| Sao Tome and Principe | 113.07 | 30.87 | 408.54 | 113.07 | High |
| Senegal | 159.71 | 115.18 | 349.76 | 159.71 | High |
| Serbia | 0.19 | 15.69 |  | 7.94 | Low |
| Sierra Leone | 194.09 | 925.15 | 496.63 | 496.63 | High |
| Solomon Islands | 146.82 | 1,428.61 | 18.29 | 146.82 | High |
| Somalia | 199.37 | 481.84 | 438.15 | 438.15 | High |
| South Africa | 2.21 | 72.46 | 176.09 | 72.46 | Medium |
| South Sudan | 126.05 | 259.85 |  | 192.95 | High |
| Sri Lanka | 152.71 | 16.27 | 295.96 | 152.71 | High |
| St Vincent & the Grenadines | 2.04 | 17.47 | 25.25 | 17.47 | Medium |
| Sudan | 47.1 | 254.39 | 24.56 | 47.1 | Medium |
| Suriname | 1.21 | 66.38 | 24.95 | 24.95 | Medium |
| Swaziland | 3.48 | 116.1 | 243.46 | 116.1 | High |
| Syrian Arab Republic | 39.45 | 18.04 | 14.47 | 18.04 | Medium |
| Tajikistan | 0.49 | 22.88 | 436.26 | 22.88 | Medium |
| Tanzania | 117.5 | 553.27 | 164.7 | 164.7 | High |
| TFYR Macedonia | 0.15 | 18.61 |  | 9.38 | Low |
| Thailand | 212.46 | 8.15 | 199.53 | 199.53 | High |
| Timor-Leste | 260.16 | 412.99 | 395.83 | 395.83 | High |
| Togo | 229.03 | 168.1 | 413 | 229.03 | High |
| Tonga | 122.29 | 91.07 | 12.54 | 91.07 | Medium |
| Tunisia | 35.89 | 25.67 | 11.66 | 25.67 | Medium |
| Turkey | 41.77 | 10.36 | 12.9 | 12.9 | Medium |
| Turkmenistan | 0.28 | 22.04 | 303.01 | 22.04 | Medium |
| Uganda | 103.83 | 146.07 | 329.42 | 146.07 | High |
| Ukraine | 0.12 | 6.69 |  | 3.41 | Low |
| Uzbekistan | 0.28 | 18.2 | 295.84 | 18.2 | Medium |
| Vanuatu | 125.01 | 201.15 | 20.32 | 125.01 | High |
| Venezuela | 0.11 | 47.71 | 31.14 | 31.14 | Medium |
| Viet Nam | 148.84 | 60.28 | 128.54 | 128.54 | High |
| Yemen | 76.21 | 5,849.85 | 22.32 | 76.21 | Medium |
| Zambia | 130.76 | 361.97 | 367.02 | 361.97 | High |
| Zimbabwe | 3.45 | 274.39 | 250.3 | 250.3 | High |
