# Supplementary material for: Forecasting Demand for the Typhoid Conjugate Vaccine in Low- and Middle-income Countries
Source: Clin Infect Dis. 2019 Mar 7;68(Suppl 2):S154–60. doi: 10.1093/cid/ciy1076 (PMC6405267; doi:10.1093/cid/ciy1076)
Supplement: Supplementary Table 2 [file ciy1076_suppl_supplement_table_2.docx]

*Supplementary table 2: Unadjusted year of introduction*

|  | **Very High disease incidence** (>500 cases per 100,000) | **High disease incidence** >50 <500 cases per 100,000 | **Medium disease incidence** < 50 cases per 100,000 | **Low disease incidence** <10 cases per 100,000 |
| --- | --- | --- | --- | --- |
| **Early adopter** |  | Ethiopia | Yemen | Moldova |
|  |  | Zambia | South Africa |  |
|  |  | Rwanda | Lesotho |  |
|  |  | Philippines | Bolivia |  |
|  |  | Angola | Nicaragua |  |
|  |  | Cameroon | Fiji |  |
|  |  | Zimbabwe | Morocco |  |
|  |  | Kenya | Honduras |  |
|  |  | Mauritania | Guatemala |  |
|  |  | Gambia | Libya |  |
|  |  | Malawi | Peru |  |
|  |  | Tanzania | Paraguay |  |
|  |  | Senegal | Venezuela |  |
|  |  | Botswana | El Salvador |  |
|  |  | Micronesia | Brazil |  |
|  |  | Sao Tome and Principe | Panama |  |
|  |  |  | Ecuador |  |
|  |  |  | Guyana |  |
|  |  |  | Colombia |  |
|  |  |  | Argentina |  |
|  |  |  | Mexico |  |
|  |  |  | Dominican Republic |  |
|  |  |  | Armenia |  |
| **Moderate adopter** | DR Congo | Sierra Leone | Sudan | Macedonia |
|  | CAR | Niger | Albania | Bulgaria |
|  | Burkina Faso | Benin | Kazakhstan |  |
|  |  | Mali | Costa Rica |  |
|  |  | Madagascar | Iraq |  |
|  |  | Liberia | Kyrgyzstan |  |
|  |  | Congo | Uzbekistan |  |
|  |  | Bhutan | Mauritius |  |
|  |  | Eritrea | Turkey |  |
|  |  | Mozambique | Jamaica |  |
|  |  | Pakistan | Georgia |  |
|  |  | Burundi |  |  |
|  |  | Togo |  |  |
|  |  | Ghana |  |  |
|  |  | Thailand |  |  |
|  |  | Namibia |  |  |
|  |  | Solomon Islands |  |  |
|  |  | Uganda |  |  |
|  |  | Kiribati |  |  |
|  |  | Malaysia |  |  |
|  |  | Swaziland |  |  |
|  |  | Djibouti |  |  |

| **Late adopter** | Afghanistan | Nigeria | Haiti | Russia |
| --- | --- | --- | --- | --- |
|  | Bangladesh | India | Belize | Belarus |
|  |  | Lao PDR | St. Lucia | Ukraine |
|  |  | Nepal | Suriname | Croatia |
|  |  | Côte d'Ivoire | Mongolia |  |
|  |  | Cambodia | Tajikistan |  |
|  |  | Guinea-Bissau | Grenada |  |
|  |  | Myanmar | Turkmenistan |  |
|  |  | Papua New Guinea | Lebanon |  |
|  |  | Sri Lanka | St. Vincent and the Grenadines |  |
|  |  | Indonesia | Jordan |  |
|  |  | Vanuatu | Azerbaijan |  |
|  |  | Samoa |  |  |
|  |  | Cape Verde |  |  |
| **Non-adopter** | Chad | Somalia | Tonga | Romania |
|  |  | Timor-Leste | Comoros | Serbia |
|  |  | Gabon | Maldives | Montenegro |
|  |  | South Sudan | Equatorial Guinea | Bosnia and Herzegovina |
|  |  | Guinea | Iran |  |
|  |  | Vietnam | Algeria |  |
|  |  |  | Tunisia |  |
|  |  |  | Egypt |  |
|  |  |  | China |  |
|  |  |  | Cuba |  |
|  |  |  | Syria |  |
|  |  |  | Korea DPR |  |
