# Supplementary material for: Forecasting Demand for the Typhoid Conjugate Vaccine in Low- and Middle-income Countries
Source: Clin Infect Dis. 2019 Mar 7;68(Suppl 2):S154–60. doi: 10.1093/cid/ciy1076 (PMC6405267; doi:10.1093/cid/ciy1076)
Supplement: Supplementary Table 3 [file ciy1076_suppl_supplement_table_3.docx]

*Supplementary table 3: model inputs*

| **Model inputs** | **Values used in the analysis** | **Rationale** |
| --- | --- | --- |
| Countries | Low, lower-middle, and upper-middle income countries | We focused on these countries as the most likely to consider implementing vaccination against typhoid fever as part of the routine infant immunization schedule. We considered high income countries likely to continue vaccinating only as needed for travelers. Income classifications were obtained from the World Bank.  We included current Gavi status and projected likely graduation dates based on trends in gross national income per capita. |
| Time horizon | 2020 to 2040 | We estimated that the first Gavi-funded introductions of TCV are likely to occur in 2020. In order to capture the period in which production capacity is most likely to be strained, we focused on the first 20 years of use. |
| Population | UN population projection, 2017 revision, medium estimates | We used the medium projection in order to estimate the most likely scenario. |
| Vaccination strategy | Routine immunization: 1 dose per child administered at 9 months  Catch-up campaign: 1 dose administered to individuals 1 to 15 years old. Catch-up campaigns are optional and may target different share of the total target population. | We modeled the vaccination strategy according to last WHO SAGE recommendation. |
| Coverage | MCV1 | MCV1 is used as a measure of country routine immunization performance and was chosen as first dose of measles containing vaccine is likely to be administered at the same age than TCV. MCV1 is a commonly used proxy for coverage of new vaccines. We chose to carry the most recent value forward to 2040 to reflect present performance. |
| Wastage | 10% in campaigns, 15% in routine | We chose these numbers to reflect the current product’s availability in five-dose vial presentation. |
| Buffer stock | 25% | Buffer stock was set at 25% to cover for unexpected interruptions in supply or sudden increase in demand. This is in accordance with WHO recommendations. |
